# Lymphoedema Development Following a Cancer Diagnosis: An Anonymised Data Linkage Study in Wales, United Kingdom

**DOI:** 10.1111/iwj.70331

**Published:** 2025-04-16

**Authors:** Ioan Humphreys, Alan Watkins, Ashley Akbari, Rowena Griffiths, Marie Gabe‐Walters, Melanie Thomas, Cheryl Pike, Angela Williams, Tom Dobbs, John Gibson, Iain S. Whitaker, Hayley A. Hutchings

**Affiliations:** ^1^ School of Health and Social Care, Faculty of Medicine, Health and Life Sciences Swansea University Swansea UK; ^2^ Swansea University Medical School, Faculty of Medicine, Health and Life Sciences Swansea University Swansea UK; ^3^ Lymphoedema UK Clinical Network Swansea Bay University Health Board UK

**Keywords:** cancer, diagnosis, linked routine data, lymphoedema, risk factors

## Abstract

This observational cohort study explored lymphoedema development following a cancer diagnosis and whether demographic factors impacted the time to lymphoedema development. We identified cases through the Secure Anonymised Information Linkage (SAIL) Databank. We used cancer diagnostic codes to identify a cohort of six broad cancer ‘types’. We independently used lymphoedema diagnostic codes to identify a cohort who developed lymphoedema. We linked these two cohorts to develop a single cohort of cases and describe the number of cases who went on to develop lymphoedema *after* a cancer diagnosis, and the time to lymphoedema diagnosis. We used Cox regression models to calculate hazard ratios and produced survival curves to explore whether pre‐defined factors (gender, age, deprivation, cancer type) had any impact on time to lymphoedema development. We identified 7538 cases of lymphoedema development after a cancer diagnosis, relating to 7279 people. There was considerable variation in the time to diagnosis, with a mean and standard deviation of 483.3 (701.8) days. Cancer type was the single most important factor in explaining time to lymphoedema diagnosis. Time to lymphoedema was shortest in breast cancer. A large number of breast cancer cases have undergone surgery, and this may account for the earlier development of lymphoedema. Consideration should be made of risk factors for lymphoedema development in order to allow for more targeted treatment plans that could improve health‐related quality of life for patients.

**Summary**

- This study had a large sample size and used data available within the SAIL Databank, which covers 84% of primary care data of the Welsh population, and 100% of secondary care data.
- A large number of individuals experience lymphoedema following a cancer diagnosis and that with some types of cancer, breast cancer in particular, this can develop within weeks of the initial diagnosis.
- Early diagnosis would also allow more targeted treatment plans and care to be put in place quicker, leading to a more cost‐effective treatment of the condition.
- Lymphoedema can develop with all cancer types and that there is a wide variation in the time to diagnosis. However, patients need to be most vigilant within the first 18‐months post diagnoses.
- All cancer healthcare professionals should have a general awareness of lymphoedema, tools for screening and knowledge surrounding the referral process enabling prompt treatment.

## 1 Background

Lymphoedema is defined as a long‐term condition that causes swelling in the body's tissues. It can affect any part of the body but usually develops in the arms or legs (https://www.nhs.uk/conditions/Lymphoedema/). It can also involve the trunk, head, or perineum [1, 2]. It develops when the lymphatic system (the system to remove excess interstitial fluid and fight infection in the body, whilst also providing a nutritional function) is not working properly, and the demand for lymphatic drainage exceeds the capacity of the lymphatic circulation [3]. Lymphoedema is a progressive condition with excess tissue oedema being the predominant feature during the early stages. As the condition advances, the limb becomes firm, tight, non‐pitting, and fibrotic, with deepened natural skin folds [4]. It causes a range of symptoms, including an aching, heavy feeling and difficulty with movement. There is an increased risk of repeated skin infections such as cellulitis, which can lead to skin changes such as wart‐like growths developing on the skin and fluid leaking through the skin (lymphorroea). It significantly impacts the lives of people who experience it and has been shown to have a detrimental effect on health‐related quality of life [1, 2, 5]. Lymphoedema can have a major physical, psychological, and social impact on patients [3, 6, 7].

Lymphoedema can be classified as primary or secondary. Secondary lymphoedema is the result of obliteration, removal, or obstruction of the lymph nodes, or from damage or obstruction of the lymphatic vessels [8]. One of the major causes of secondary lymphoedema is following treatment for cancer, in particular when the lymph nodes are removed following surgery [9]. Figures from the UK (https://www.nhs.uk/conditions/Lymphoedema/) suggest that there are around 400,000 people affected by lymphoedema (https://www.lymphoedema.org/index.php/information‐and‐support/what‐is‐lymphoedema). In Wales, the prevalence in 2024 was demonstrated as 7.2 per 1000 people, equating to 25,000 people in Wales living with lymphoedema with an incidence of 3 per 1000 [10]. As cancer incidence is increasing, this figure will increase further. The prevalence of cancer‐related lymphoedema has been suggested to be upwards of 25% [11, 12, 13].

Although survival rates for cancers are increasing, treatment‐associated morbidity is common and can persist well beyond the treatment period [14]. Paradoxically, it is the improvement in survival and the increasingly successful outcomes of oncological therapy that have led to an increase in the incidence of lymphoedema and its associated burden [1]. It has been suggested that lymphoedema develops in approximately one‐fifth of cancer survivors, with incidence increasing over time [14]. From an economic perspective, the treatment of chronic lymphoedema is becoming more common, with the therapeutic regime often being complex, time‐consuming, and requiring constant and steady treatment and self‐management regimes [2, 15, 16]. In addition, lymphoedema is associated with a high rate of complications such as chronic wounds and cellulitis [9, 17, 18]. For this reason, lymphoedema leads to high costs and disease burden. A 2017 European study found that the average cost per patient was almost €6000 [17] and this has almost certainly increased further since that time.

Given the increasing number of patients being diagnosed and successfully treated for cancer, and the growing number of patients experiencing lymphoedema, there is a need to understand more about this complex disease to promote early intervention.

There is a developing body of research aimed at identifying, preventing, and treating lymphoedema, including exploration of potential risk factors that may contribute to the development of lymphoedema. Most of this work has focussed on patients undergoing surgical treatment for breast cancer [9, 14, 19, 20]. A 2019 review estimated that 1 in 6 women treated for breast cancer will develop lymphoedema within months to years after diagnosis and treatment [21] and a 2020 systematic review [12] similarly identified arm‐related lymphoedema as the most reported symptom after breast cancer treatment. A 2008 Australian population‐based study reported that oedema presented 6 to 18 months after surgery for invasive breast cancer in 33% of women, with 40% identified as having lymphoedema [22]. A recent systematic review estimates that prevalence rates could be as high as 74% [23].

Other cancer types (gynaecological, urological, melanoma etc.) also carry a risk of lymphoedema development depending on the extent of the tumour and cancer treatment intensity. A recent study identified that 34% of endometrial, 35% of cervical, and 43% of vulvar patients experienced lymphoedema [24]. Two studies identified that greater than 50% and greater than 75% of patients developed lymphoedema secondary to head or neck cancer treatment [25, 26]. A recent systematic review identified much higher rates with wide variation in the reported incidence of other cancer‐related lymphoedema, reporting 8%–45% for gynaecological and urological cancer, 7%–90% in head and neck cancer, and 2%–29% in melanoma cancers [23]. The body of literature, particularly in relation to other cancer types, is still limited.

This study aimed to describe the number of cases in Wales diagnosed with breast, skin, head and neck, bladder, female gynaecological, and male genitalia/prostate cancers; the time duration to lymphoedema development following a cancer diagnosis; and whether demographic information had any impact on the time to lymphoedema development.

## 2 Method

We utilised anonymised routinely collected individual‐level, population‐scale electronic health record (EHR) data sources available within the Secure Anonymised Information Linkage (SAIL) Databank to undertake this study (https://saildatabank.com) [27, 28, 29]. The SAIL Databank is the national Trusted Research Environment (TRE) for Wales, a privacy‐protecting TRE containing data including primary care events from 84% of general practices around Wales (population coverage ~3.2 million people) and secondary care in‐patient hospital episodes from 100% of National Health Service (NHS) Wales. All data are anonymised within SAIL, but individual‐level linkage is possible through an encrypted anonymised linking field which allows associations to be made between data sources and longitudinal patient pathways for analyses. We designed and reported our study in accordance with the Reporting of studies Conducted using Observational Routinely collected health Data (RECORD) statement [30].

For our study, we had data coverage from 1st January 2010 to 31st December 2020. Table 1 gives details of the specific data sources approved for use by our study within the SAIL Databank and that were used for the cohort selection and outcome identification.

**TABLE 1:** Specific data sources accessed from SAIL for the study.

Sail data source	Data source full name	Data source link
ADDE	Annual District Death Extract	https://www.ons.gov.uk/peoplepopulationandcommunity/birthsdeathsandmarriages/deaths/datasets/deathsregisteredinenglandandwalesseriesdrreferencetables
PEDW	Patient Episode Database for Wales	https://web.www.healthdatagateway.org/dataset/4c33a5d2‐164c‐41d7‐9797‐dc2b008cc852
WDSD	Welsh Demographic Service Dataset	https://web.www.healthdatagateway.org/dataset/cea328df‐abe5‐48fb‐8bcb‐c0a5b6377446
WLGP	Welsh Longitudinal General Practice	https://web.www.healthdatagateway.org/dataset/33fc3ffd‐aa4c‐4a16‐a32f‐0c900aaea3d2
WCSU	Welsh Cancer Intelligence and Surveillance Unit	https://phw.nhs.wales/services‐and‐teams/welsh‐cancer‐intelligence‐and‐surveillance‐unit‐wcisu/

Two patient cohorts (a cancer cohort of pre‐specified cancers and a lymphoedema cohort) were developed and subsequently merged to undertake analysis.

### 2.1 Cancer Cohort

We developed a cancer cohort by identifying all patients with a diagnosis of any of six pre‐specified cancer types (breast, skin, head and neck, bladder, female gynaecological, and male genitalia/prostate) registered within either the Welsh Cancer Intelligence and Surveillance Unit (WCSU) or Patient Episode Database for Wales (PEDW) data sources between 1st January 2010 and 31st December 2020. At the time of our study, WCSU updates were suspended due to the COVID‐19 pandemic (mid 2020), so we identified any new cancer cases after this date using PEDW data only. We identified the date of cancer diagnosis as the first admission recorded for each respective cancer. International Classification of Diseases version 10 (ICD‐10) codes were used to identify each cancer type of interest (breast, skin, head and neck, bladder, female gynaecological, and male genitalia/prostate) in PEDW and WCSU. Details of the specific codes used are documented in Table S1.

### 2.2 Lymphoedema Cohort

We developed a lymphoedema cohort by identifying patients with a diagnosis of lymphoedema in either PEDW or WLGP data sources. We used the earliest date recorded in either PEDW or WLGP as the date of diagnosis for lymphoedema. We used ICD‐10 and Read codes version 2 to identify lymphoedema in WLGP and PEDW. Details of the specific codes used are documented in Table S2.

### 2.3 Combined Cohort

Following the development of the two patient cohorts (cancer and lymphoedema), we undertook data matching using their Anonymised Linking Field (ALF) [29] to create a single cohort of cases with both cancer and lymphoedema. We also collected data from the WDSD to gather basic demographic information (age, sex). We used the Welsh Index of Multiple Deprivation (WIMD; https://www.gov.wales/welsh‐index‐multiple‐deprivation) version 2014 quintile to measure area‐level socio‐economic status and deprivation assigned based on each individual's Lower‐layer Super Output Area (LSOA) of residence version 2011.

### 2.4 Statistical Methods

We used SPSS (Version 25) to analyse an integrated study dataset, created using SQL via Eclipse from data accessed within the SAIL Databank using IBM DB2.

We defined our primary variable as the time (in days) between cancer and lymphoedema diagnoses. We allocated patients to age bands (using age at cancer diagnosis) and grouped cancers into 6 broad classes (‘types’), with a further simplification into ‘breast’ and ‘other’.

We obtained summaries of the primary variable across a range of factors—gender (female/male); age band; cancer location; and WIMD quintile—and supplemented these with Kaplan–Meier survival curves. We then used Cox regression models to assess the relationship between our primary variable and this set of explanatory factors, both individually and collectively.

## 3 Results

### 3.1 Cancer and Lymphoedema Cohorts

We identified 107,649 people with one of the pre‐defined cancers. The mean age of the cancer diagnosis cohort was 63.4 years, with a range from 11 to 100 years.

We identified 26,843 patients with a lymphoedema diagnosis. The mean age of the lymphoedema diagnosis cohort was 64.8 years, with a range from 11 to 102 years.

### 3.2 Combined Cohort

We identified 9131 cases of *both* a cancer diagnosis *and* a lymphoedema diagnosis, with 7538 cases where the lymphoedema developed *after* the cancer diagnosis. These 7538 cases related to 7279 unique people, who form our combined cohort. Table 2 summarises their demographic characteristics. Most cases were female. The most common age categories were 46–60 and 61–75 years, and breast cancer was the most common cancer identified.

**TABLE 2:** Demographic characteristics of the combined cohort (*n* = 7279).

Factor	*n* (%)
Gender	
Female	6148 (84.5%)
Male	1131 (15.5%)
Age group (years)	
11–30	63 (0.9%)
31–45	637 (8.8%)
46–60	2287 (31.4%)
61–75	2902 (39.9%)
76–90	1341 18.4(%)
91+	49 (0.7%)
WIMD quintile (*n* = 7058)	
1 (most deprived)	1146 (16.2%)
2	1390 (19.7%)
3	1412 (20.0%)
4	1430 (20.3%)
5 (least deprived)	1680 (23.8%)
Cancer type	
Breast	4874 (67.0%)
Skin	1051 (14.4%)
Head & neck	334 (4.6%)
Bladder	100 (1.4%)
Female specific	575 (7.9%)
Male specific	345 (4.7%)
Cancer type	
Breast	4874 (67.0%)
‘Other’	2405 (33.0%)

### 3.3 Distributions of Times Between Cancer and Lymphoedema Diagnoses

**FIGURE 1:**
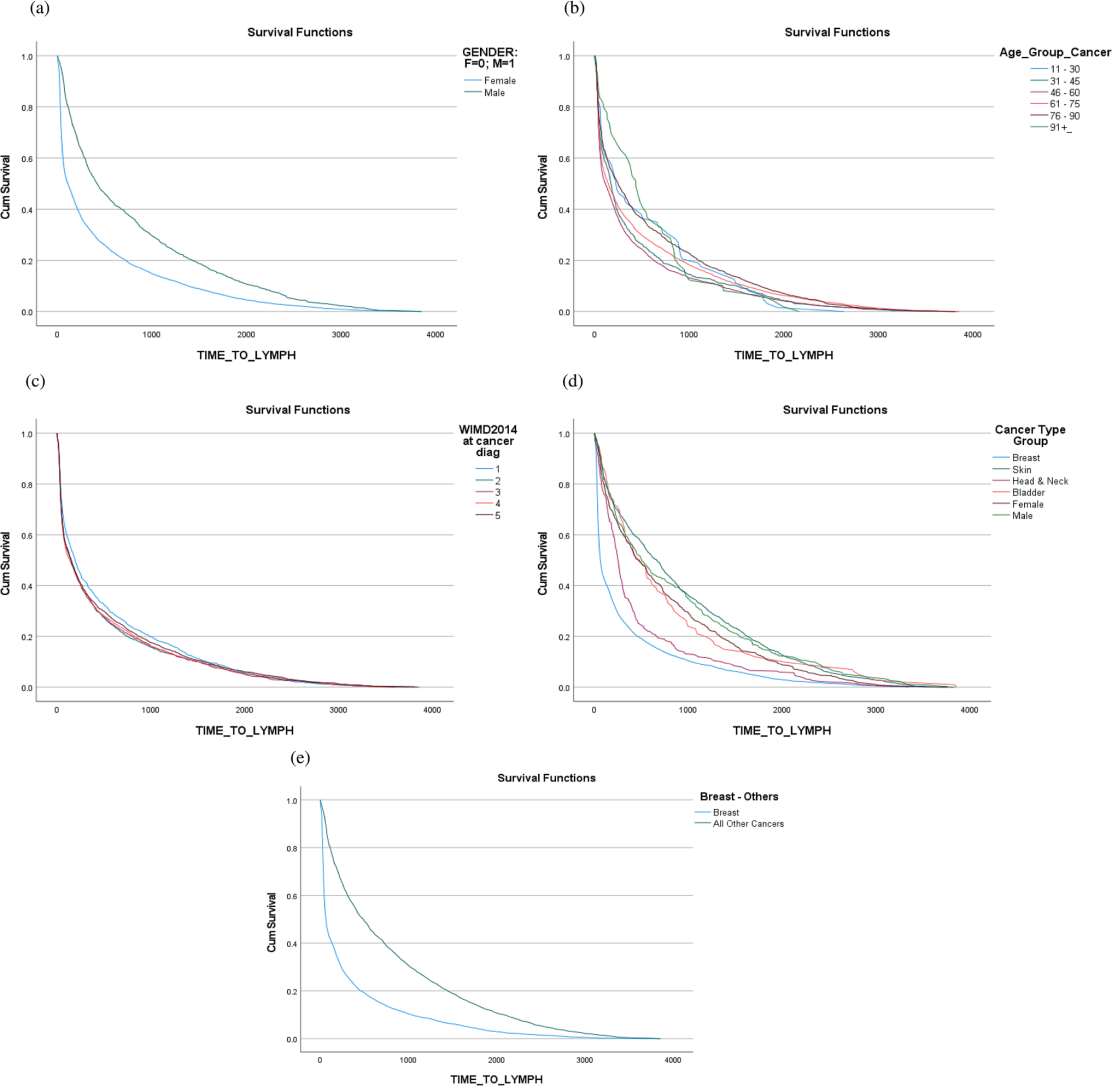
Survival curves for the time between diagnosis and our pre‐defined factors. (a) Survival curves for time between diagnoses, by gender. (b) Survival curves for the time between diagnoses by age group. (c) Survival curves for time between diagnoses by WIMD quintile. (d) Survival curves for the time between diagnoses, by cancer type. (e) Survival curves for time between diagnoses for ‘breast’ and ‘non‐breast’ cancers.

Across the combined cohort, the mean time between cancer and lymphoedema diagnoses was 483.3 (days), with considerable variation around this value; the standard deviation of 701.8 (days) indicates significant skewness. Table 3, therefore, summarises the distribution of this variable across each factor using the 25th, 50th and 75th percentiles—that is, the median and lower and upper quartile—with a formal comparison via hazard ratios from Cox regression models, supported by survival curves in Figure 1a–e.

**TABLE 3:** The distributions of times between diagnoses in days, by factor.

Factor	Time between diagnoses (percentiles)			Hazard ratio (unadjusted)			Figure
	25th	50th	75th	Estimate	*p*	(95% CI)	
Gender							
Female	34	108	530	*Reference*			1(a)
Male	147	419	1179	0.621	< 0.001	(0.583, 0.662)	
Age (years)							
All	n/a	n/a	n/a	0.994	< 0.001	(0.992, 0.996)	n/a
Age group (years)							
11–30	60	224	896	n/a	n/a	n/a	1(b)
31–45	39	169	544				
46–60	35	111	470				
61–75	37	143	698				
76–90	47	244	891				
91+	141	432	835				
WIMD quintile							
1	43	190	742	*Reference*			1(c)
2	38	149	567	1.086	0.038	(1.005, 1.175)	
3	36	150	579	1.094	0.023	(1.012, 1.183)	
4	36	134	618	1.097	0.019	(1.015, 1.186)	
5	38	147	672	1.055	0.164	(0.978, 1.137)	
Cancer type							
Breast	31	62	330	*Reference*			
Skin	172	662	1428	0.455	< 0.001	(0.425, 0.487)	1(d)
Head & neck	116	252	483	0.700	< 0.001	(0.627, 0.782)	
Bladder	159	480	991	0.471	< 0.001	(0.386, 0.575)	
Female specific	134	469	1141	0.524	< 0.001	(0.481, 0.572)	
Male specific	175	502	1333	0.462	< 0.001	(0.414, 0.515)	
Cancer type							
Breast	31	62	330	*Reference*			1(e)
‘Other’	149	483	1220	0.498	< 0.001	(0.474, 0.523)	

Comparison of time to lymphoedema diagnosis identified that ‘other’ cancers had much longer times to diagnosis when compared with breast cancer. Cases with breast cancer developed lymphoedema a median of 2 months after their cancer diagnosis (upper and lower quartile, 12 to 12 months). Cases with ‘other’ cancers developed lymphoedema a median of 16 months (upper and lower quartile, 5 to 39 months).

### 3.4 Full Cox Regression Models

Figure 1 implies that all factors are potentially useful in explaining observed variation in time between diagnoses. We therefore fitted a sequence of Cox regression models, starting with a full model including all factors, and then removing any non‐significant factors until only statistically significant factors remained.

This process is summarised in Table 4, which shows that cancer type is the single most useful explanatory factor. The final model in Table 4 (Model 4) is as shown in Table 3, with hazard ratios obtained by exponentiating the coefficients.

**TABLE 4:** Coefficients from multivariate Cox regression models.

Variable		Model			
		1 (Full)	2	3	4 (Final)
Gender	Coefficient (*p*‐value)	0.030 (0.552)	Omitted	Omitted	Omitted
Cancer type group (1)	Coefficient (*p*‐value)	−0.812 (< 0.001)	−0.798 (< 0.001)	−0.801 (< 0.001)	−0.787 (< 0.001)
Cancer type group (2)	Coefficient (*p*‐value)	−0.376 (< 0.001)	−0.358 (< 0.001)	−0.357 (< 0.001)	−0.357 (< 0.001)
Cancer type group (3)	Coefficient (*p*‐value)	−0.781 (< 0.001)	−0.762 (< 0.001)	−0.765 (< 0.001)	−0.753 (< 0.001)
Cancer type group (4)	Coefficient (*p*‐value)	−0.652 (< 0.001)	−0.652 (< 0.001)	−0.650 (< 0.001)	−0.646 (< 0.001)
Cancer type group (5)	Coefficient (*p*‐value)	−0.798 (< 0.001)	−0.768 (< 0.001)	−0.782 (< 0.001)	−0.782 (< 0.001)
WIMD2014 quintile	Coefficient (*p*‐value)	0.010 (0.258)	0.009 (0.264)	Omitted	Omitted
Age (years)	Coefficient (*p*‐value)	0.001 (0.189)	0.001 (0.190)	0.001 (0.166)	Omitted

## 4 Conclusion

This is the first attempt to describe the numbers of cases of lymphoedema in Wales following a cancer diagnosis using the SAIL Databank. Across our 10‐year period, 7538 cancer types were identified for 7279 distinct individuals. The mean age at cancer diagnosis was 63.4 years, with the mean age at lymphoedema diagnosis of 64.8 years. This means, on average, that lymphoedema tends to develop around 12 months after diagnosis. Most cases were female. The most common age categories were 46–60 and 61–75 years, and breast cancer was the most common cancer identified. There was considerable variation between the mean time to diagnosis of lymphoedema in our combined cohort of 483.3 days, with a standard deviation of 701.8 days. Initial analysis of the pre‐defined factors (sex, age, deprivation, cancer type) indicated that they could all be useful in explaining the time to lymphoedema diagnosis. However, when further analysis was undertaken using Cox regression models, only cancer type was the single most explanatory factor. Comparison of time to lymphoedema diagnosis identified that ‘other’ cancers had much longer times to diagnosis when compared with breast cancer.

Our findings mirror those of a 2019 review which identified that 1 in 6 women treated for breast cancer will develop lymphoedema within months to years after diagnosis and treatment [21]. The high number of cases of lymphoedema following a breast cancer diagnosis could be due to the fact that there is an increasing incidence of breast cancer, with more patients undergoing breast surgery [31]. In addition, there is likely to be increased vigilance and standard referral pathways for breast cancer patients to lymphoedema services which may not exist or be formalised for ‘other’ cancer types. Chest wall radiotherapy is also commonly performed in breast cancer patients, and both surgery and radiotherapy can cause lymphedema, with significant impairment of the normal lymphatic drainage producing an abnormal collection of protein‐rich fluid within the upper limb [31]. Similarly, the earlier median onset time of lymphoedema in breast cancer cases of 2 months aligns with previous research indicating that lymphoedema can develop within days postoperatively, with an increased prevalence over time [32].

Other studies have found estimates of varying incidence of lymphoedema ranging from 2% to 83% in various cancers [22, 23, 33, 34, 35]. Most of these have indicated increased incidence of breast cancer when compared with other cancer types [23, 33, 36]. Many risk factors for the development of lymphoedema have been identified depending on the site of the cancer and include a diagnosis of breast cancer, advanced cancer stage, types of surgery, radiotherapy, and being overweight to name but a few [37].

This study had a large sample size and used data available within the SAIL Databank, which covers 84% of primary care data of the Welsh population and 100% of secondary care data. Extrapolations made from these data are likely to represent a realistic estimate of the problem. Although clinical coding may be an issue, we have tried to improve data quality using pre‐specified codes for our cancers of interest and lymphoedema codes. We only explored a limited number of factors and specific cancer types in this study, so more work is needed to determine whether other factors may be significant predictors of time to lymphoedema development in addition to cancer type.

The results illustrate that a large number of individuals experience lymphoedema following a cancer diagnosis and that, with some types of cancer—breast cancer in particular—this can develop within weeks of the initial diagnosis. Lymphoedema is an expensive problem for the NHS [9, 17, 18, 38], can result in substantial discomfort, and have a detrimental impact on health‐related quality of life [1, 2, 5]. More work is needed to define risk factors that could be used to inform treatment strategies for patients with cancer. Initiatives to identify early signs/risks of lymphoedema after cancer diagnosis would greatly improve the health‐related quality of life of such patients. Early diagnosis would also allow more targeted treatment plans and care to be put in place quicker, leading to a more cost‐effective treatment of the condition.

## Ethics Statement

We received approval for the use of anonymised data within the SAIL Databank from the SAIL independent Information Governance Review Panel (IGRP) under project 0969. The IGRP provides independent guidance and advice on information governance policies, procedures and processes for SAIL Databank (https://saildatabank.com/governance/approvals‐public‐engagement/information‐governance/). Data held within the SAIL Databank are made available to researchers in an anonymised format and are therefore not subject to data protection legislation. SAIL follows all relevant legislative and regulatory frameworks in using population data for research.

## Conflicts of Interest

The authors declare no conflicts of interest.

## Supporting information


**Data S1.** Supporting Information.

## Data Availability

The data used in this study are available in the SAIL Databank at Swansea University, Swansea, UK, but as restrictions apply they are not publicly available. All proposals to use SAIL data are subject to review by an IGRP. Before any data can be accessed, approval must be given by the IGRP. The IGRP gives careful consideration to each project to ensure proper and appropriate use of SAIL data. When access has been granted, it is gained through a privacy protecting safe haven and remote access system referred to as the SAIL Gateway. SAIL has established an application process to be followed by anyone who would like to access data via SAIL at https://www.saildatabank.com/application‐process.
