# Supplementary material for: Lymphoedema Development Following a Cancer Diagnosis: An Anonymised Data Linkage Study in Wales, United Kingdom
Source: Int Wound J. 2025 Apr 16;22(4):e70331. doi: 10.1111/iwj.70331 (PMC12003098; doi:10.1111/iwj.70331)
Supplement: Supplementary file 1 — Data S1. Supporting Information. [file IWJ-22-e70331-s001.docx]

**Table 1. Specific data sources accessed from SAIL for the study.**

| SAIL Data source | Data source full name | Data source link |
| --- | --- | --- |
| ADDE | Annual District Death Extract | <https://www.ons.gov.uk/peoplepopulationandcommunity/birthsdeathsandmarriages/deaths/datasets/deathsregisteredinenglandandwalesseriesdrreferencetables> |
| PEDW | Patient Episode Database for Wales | https://web.www.healthdatagateway.org/dataset/4c33a5d2-164c-41d7-9797-dc2b008cc852 |
| WDSD | Welsh Demographic Service Dataset | <https://web.www.healthdatagateway.org/dataset/cea328df-abe5-48fb-8bcb-c0a5b6377446> |
| WLGP | Welsh Longitudinal General Practice | <https://web.www.healthdatagateway.org/dataset/33fc3ffd-aa4c-4a16-a32f-0c900aaea3d2> |
| WCSU | Welsh Cancer Intelligence and Surveillance Unit | <https://phw.nhs.wales/services-and-teams/welsh-cancer-intelligence-and-surveillance-unit-wcisu/> |

**Table 2. Demographic characteristics of the combined cohort (n=7279).**

| **Factor** | | **n** | **(%)** |
| --- | --- | --- | --- |
| **Gender** | Female | 6148 | (84.5%) |
|  | Male | 1131 | (15.5%) |
| **Age Group**  **(years)** | 11-30 | 63 | (0.9%) |
|  | 31-45 | 637 | (8.8%) |
|  | 46-60 | 2287 | (31.4%) |
|  | 61-75 | 2902 | (39.9%) |
|  | 76-90 | 1341 | 18.4(%) |
|  | 91+ | 49 | (0.7%) |
| **WIMD quintile (n=7058)** | 1 (most deprived) | 1146 | (16.2%) |
|  | 2 | 1390 | (19.7%) |
|  | 3 | 1412 | (20.0%) |
|  | 4 | 1430 | (20.3%) |
|  | 5 (least deprived) | 1680 | (23.8%) |
| **Cancer type** | Breast | 4874 | (67.0%) |
|  | Skin | 1051 | (14.4%) |
|  | Head & neck | 334 | (4.6%) |
|  | Bladder | 100 | (1.4%) |
|  | Female specific | 575 | (7.9%) |
|  | Male specific | 345 | (4.7%) |
| **Cancer type** | Breast | 4874 | (67.0%) |
|  | ‘Other’ | 2405 | (33.0%) |
